# Development of the Mammalian Expression Vector System that can be Induced by IPTG and/or Lactose

**DOI:** 10.4014/jmb.2003.03030

**Published:** 2020-05-08

**Authors:** Seung-Hyun Myung, Junghee Park, Ji-Hye Han, Tae-Hyoung Kim

**Affiliations:** Department of Biochemistry and Molecular Biology, Chosun University School of Medicine, 309 Pilmoon-Daero, Dong-Gu, Gwang-Ju 61452, Republic of Korea

**Keywords:** Inducible vector, IPTG-responsive promoter, mammalian inducible plasmid

## Abstract

Techniques used for the regulation of gene expression facilitate studies of gene function and treatment of diseases via gene therapy. Many tools have been developed for the regulation of gene expression in mammalian cells. The Lac operon system induced with isopropyl β-D-1- thiogalactopyranoside (IPTG) is one of the employed inducible systems. IPTG mimics the molecular structure of allolactose and has a strong affinity for the corresponding repressor. IPTG is known to rapidly penetrate into mammalian cells and exhibits low toxicity. In the present study, we developed a new inducible expression system that could regulate the expression of genes in mammalian cells using IPTG. Here we confirm that unlike other vector systems based on the Lac operon, this expression system allows regulation of gene expression with lactose in the mammalian cells upon transfection. The co-treatment with IPTG and lactose could improve the regulatory efficiency of the specific target gene expression. The regulation of gene expression with lactose has several benefits. Lactose is safe in humans as compared to other chemical substances and is easily available, making this technique very cost-effective.

## Introduction

Regulation of gene expression is essential in various fields such as cancer therapy, cardiovascular disorders, and genetic diseases as well as for the study of gene functions [1, 2]. Many techniques unique to mammalian cells and several different types of vehicles have been developed to regulate gene expression. Viruses and plasmid DNAs are the commonly used vehicles to regulate gene expression. Viruses exhibit high infection efficiency [2, 3] but are difficult to handle owing to the risk of infection and may affect the genes of the infected cells[2-4]. While plasmids are easy to handle, it is difficult to control gene expression. Therefore, inducible gene expression systems may be used depending on the purpose of the experiment.

Several tools have been employed to regulate gene expression in mammalian cells. Gene regulation could be accomplished with either silencing or overexpression of genes in mammalian cells. The Cre-LoxP recombination system and RNA interference (RNAi) are commonly used methods for silencing the expression of genes of interest. The Cre-LoxP recombination system is one of the most commonly used gene regulation system derived from the bacteriophage P1 [5]. The Cre recombinases recognize the LoxP site and delete specific regions surrounded by a pair of LoxP site via homologous recombination [5, 6]. However, this process is irreversible because the deleted target sequence may not be restored. Furthermore, this system requires perpetual expression of Cre recombinase, which could cause other genetic issues. However, the development of an inducible system would allow Cre recombinase expression only for a short period of time as per our need. RNAi is also commonly used to silence the expression of target genes. RNAi is characterized with the introduction of a microRNA or double-stranded RNA such as a small-interfering RNA (siRNA) that is complementary to the sequence of the target mRNA in mammalian cells [7-9]. After its introduction into mammalian cells, the siRNA or microRNA binds and subsequently cleaves the target mRNA, thereby silencing the targeted gene [8, 9]. Disadvantages of this system include its unsuitability for gene overexpression and the possibility of off-target effects [10]. Another way to regulate gene expression is through gene overexpression. The Lac operon system is one of the most commonly used system to regulate gene expression. This system was first identified in *Escherichia coli* and has been commonly used to regulate the expression of genes in bacteria [11]. In recent years, the applicability of this system has been extended in mammalian cells to induce the overexpression of target genes with the inducer, isopropyl β- D-1-thiogalactopyranoside (IPTG) [12-16].

IPTG, doxycycline, and tetracycline are some of the most commonly used inducers for mammalian cells [16- 19]. The regulation of gene expression using these inducers and specific inducer-response elements offers great advantages. These methods may allow induction of target gene expression in mammalian cells as per our requirement. IPTG is a promising inducer, as it is less toxic than other inducers such as doxycycline and tetracycline [20, 21] and exhibits high affinity (Ka = 106 M−1) [22]. In addition, IPTG rapidly penetrates into mammalian cells [23] and irreversibly binds to the Lac repressor, leading to the induction of target gene expression. Although the Lac operon system offers these advantages, there are some associated limitations. The protein of interest may leak under non-inducing conditions [16] and affect the interpretation of results.

The number and location of Lac operators are important for the regulation of gene expression in *E. coli* [15, 24]. The most important Lac operator is located between the promoter and transcription start point [25]. The Lac repressor is bound to this Lac operon and prevents the binding between the promoter and RNA polymerase II [25]. Other two Lac operators are located in 3′ upstream region from the Lac operon promoter [24, 25]. Lac repressors bind to Lac operators and form tetramers, which create a DNA loop between Lac operators [26]. These two Lac operators are also important to regulate Lac operon expression in *E. coli*. We thought that the mutations of the five Lac operators may affect their binding to the Lac repressors and consequently inhibit the target gene expression. We designed an inducible vector to regulate the expression of important genes in mammalian cells with five Lac operators to address the limitations related with previous systems. In the present study, we designed an inducible vector for the regulation of gene expression in mammalian cells. Further, we observed that lactose could serve as an inducer for our system and improve the efficiency of our inducible vector upon co-treatment with IPTG.

## Materials and Methods

### Cell Culture and Transfection

HEK293, HeLa, and B16F10 cells were obtained from the Korean Cell Line Bank and maintained in Dulbecco’s modified Eagle’s medium (DMEM) containing 5% fetal bovine serum (FBS) (Merk, USA) and 1% penicillin- streptomycin (Corning, USA), ciprofloxacin (Fluka, USA), and gentamycin (Duchefa Biochemie, The Netherlands). All three cell lines were cultured in a 5% CO2 incubator at 37°C. HEK293, HeLa and B16F10 cells were plated in T75 flasks and transfected with 20 μg of control vector or inducible vector using 60 μg of polyethylenimine (PEI, molecular weight 4,000) (Poly Science, USA).

### Induction of GFP Expression

Before GFP expression induction, the transfected cells were plated in 24-well plates and treated with isopropyl β-D-1-thiogalactopyranoside (IPTG) (Sigma-Aldrich, USA) and/or lactose (Sigma-Aldrich) at indicated concentrations. The cells were analyzed for GFP expression under a confocal microscope.

### Fluorescence Microscopy

Transfected cells were seeded into 24 well plates (1 × 10^5^ cells/well). After treatment with the inducers, IPTG and/or lactose, the nuclei were stained with Hoechst dye at indicated time points. The cells were washed with phosphate-buffered saline (PBS) and fixed with 4% paraformaldehyde in PBS for 10 min at room temperature. Cells were washed again with PBS and incubated in PBS in 24-well plates to evaluate GFP expression and Hoechst staining under an Olympus FluoView FV1000 confocal laser scanning microscope (Olympus, Shinjuku Monolith, 2-3-1, Japan)

### Western Blot Analysis

For immunoblotting analysis, the cells transfected with control and inducible vectors were seeded in 60 mm plates. After attachment, the cells were treated with the inducers, IPTG and/or lactose, at indicated concentrations. The cells were harvested at 0 and 24 h using radioimmunoprecipitation assay (RIPA) buffer (50 mM Tris-HCl pH 7.4, 1% NP-40, 0.5% Na-deoxycholate, 0.1% sodium dodecyl sulfate [SDS], 150 mM sodium chloride [NaCl], 2 mM ethylenediaminetetraacetic acid [EDTA], and 1/1000 protease inhibitor cocktail, Sigma-Aldrich). Protein concentrations were quantified with the bicinchoninic acid (BCA) assay method (ThermoFisher Scientific, USA). About 30 μg of proteins were separated with SDS polyacrylamide gel electrophoresis and the separated protein bands were transferred onto polyvinylidene fluoride (PVDF) membranes (GE Healthcare Life Science, USA) using an electro-transfer machine at 100 mA for 400 min. The membranes were blocked with 5% skim milk (BD; Becton, Dickinson and Company, USA) for 1 h and incubated with primary antibodies against GFP (SantaCruz, USA) and glyceraldehyde 3-phosphate dehydrogenase (GAPDH)(Cell Signaling, USA) for 2 h, followed by washing thrice with Tris-buffered saline-0.05% Tween-20 (TBST) buffer for 15 min. The membranes were probed with a secondary goat anti-rabbit IgG (1:3000 dilution in TBST buffer) (ThermoFisher Scientific) for 1 h and then washed four times with TBST buffer for 15 min. The membranes were developed with chemiluminescent horseradish peroxidase substrates (Millipore, USA).

## Results

### The Design of An Inducible Vector System

**Fig. 1:**
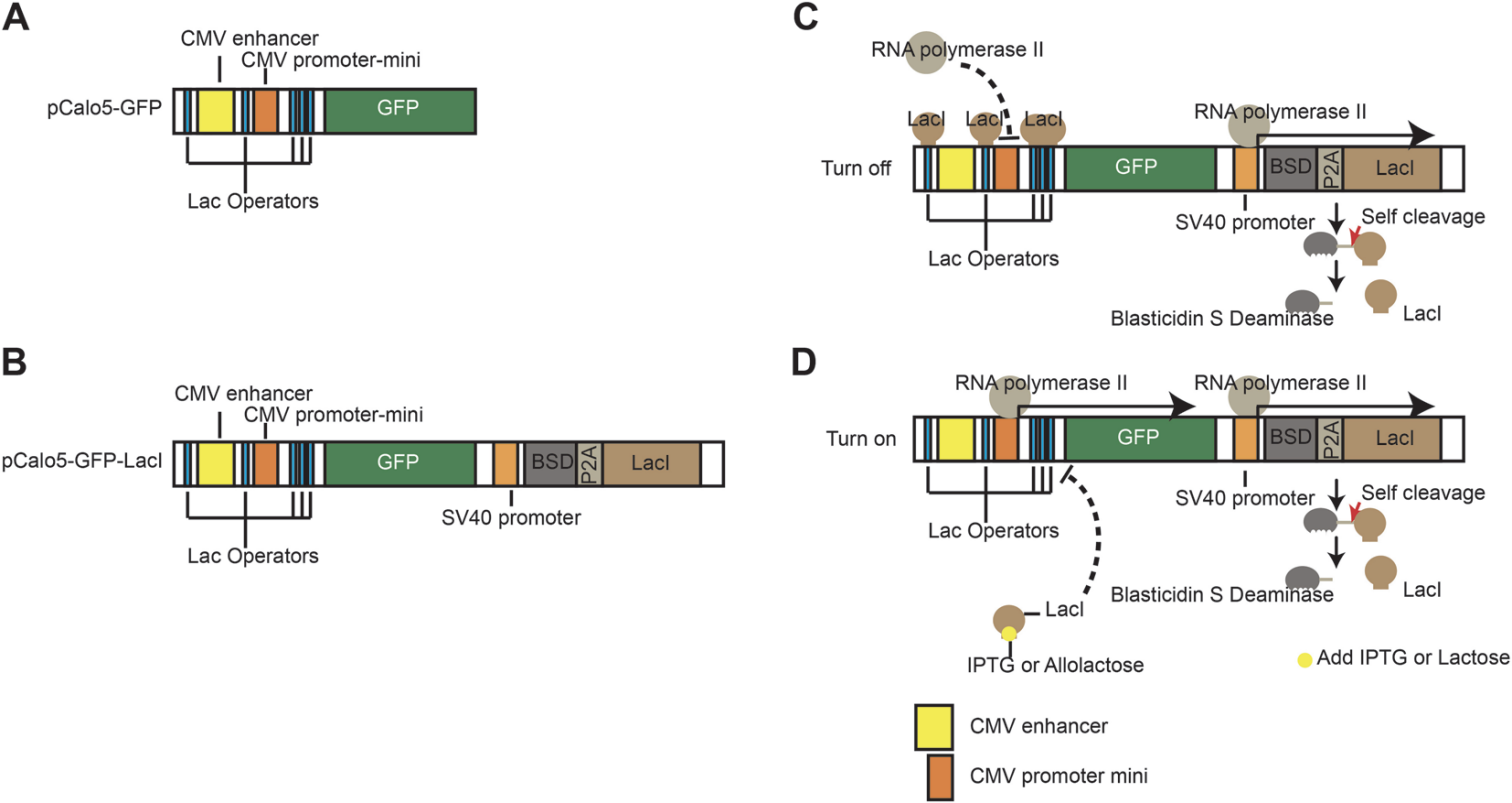
The scheme of inducible vector, pCalo5-GFP-LacI. (**A**) The control vector, pCalo5-GFP against pCalo5-GFP- LacI, (**B**) The inducible vector, pCalo5-GFP-LacI has five operators, Blasticidin gene, P2A gene and LacI gene. Three operators are located between CMV promoter and GFP ORF. The other operator is located between the promoter and the enhancer, and another one is located upstream from the enhancer. (**C**)The GFP expression is suppressed by inhibition of repressors when the inducers are not treated. (**D**)When the inducers are treated, repressors are bind to inducers such as IPTG. After that, The GFP can be expressed by transcription factors.

The location and number of Lac operators are important in the Lac operon system to regulate the expression of target genes [12-14]. Here, we developed a vector system for the regulation of gene expression in animal cells based on the results of previous studies. We first designed a vector with five Lac operators, a CMV enhancer, and a CMV promoter-mini, which could strongly induce the expression of GFP used as the reporter (Fig. 1A). CMV promoter-mini is a sequence corresponding to the minimized size of a functional promoter of mammalian cells [27]. Five Lac operators could inhibit GFP expression in the presence of the simultaneous expression of Lac repressors in the same cell. The location of each operator was as follows: One operator was located in the 5′ upstream region from the CMV enhancer, while another operator was located between the CMV enhancer and CMV promoter-mini. The other three operators were located between CMV promoter-mini and the transcription start point of GFP (Figs. 1A and B). We constructed the inducible vector by inserting *LacI* gene fused to the gene encoding blasticidin S deaminase (BSD) and *P2A* gene. The SV40 promoter could strongly induce the transcription of *LacI* gene in mammalian cells. We termed this inducible vector as pCalo5-GFP-LacI (Fig. 1B). The LacI repressor gene was fused to blasticidin gene and *P2A*, which is a protease that can cleave its C-terminal site. After the expression of *LacI* gene, the LacI repressor fused with blasticidin and P2A was cleaved by P2A, owing to its self-cleavage activity [28]. Therefore, we could select the transfected cells with pCalo5-GFP-LacI vector using blasticidin (Fig. 1B). The LacI repressor could bind to Lac operators on the regulatory region of pCalo5-GFP-LacI and inhibit GFP expression in mammalian cells (Fig. 1C). However, GFP could be expressed upon treatment with the inducer, IPTG (Fig. 1D). Therefore, we expect that the mammalian cells transfected with this vector show no transcription of the target gene until being induced with IPTG.

### Regulation of Gene Expression with IPTG or Lactose

**Fig. 2:**
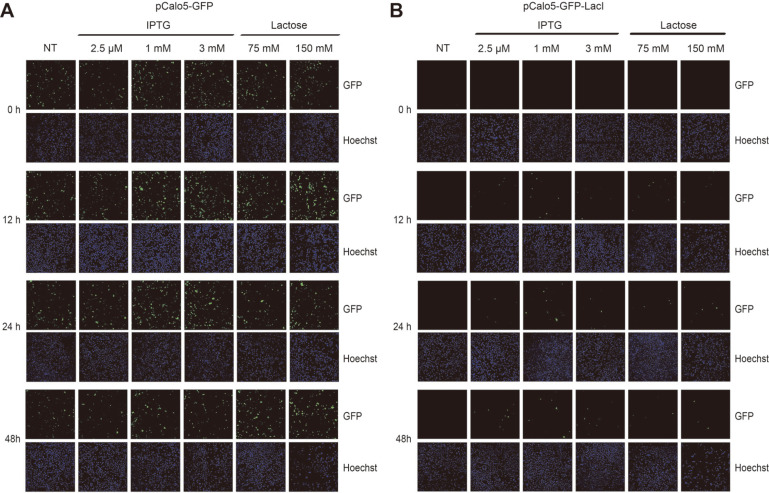
The expression of GFP using IPTG or lactose in HEK293 cell line. Transfected HEK293 cells were seeded into 24 well plates (1 × 105 cells/well) before induction of gene expression. Various concentrations of IPTG or lactose were treated into transfected HEK293 cells for the indicated time. After indicated time, 10 μg/ml of Hoechst was treated into each well for 10 min and 4% Paraform-aldehyde was treated into each well for fixation of cells for 10 min. The green color represents expressed GFP in HEK293 cells and the blue color represents nucleus stained by Hoechst.

The Lac operon system uses lactose as an energy source under low glucose environment in *E. coli*. Allolactose is an inducer that is transformed from lactose by the enzyme β-galactosidase and induces the expression of *lacZ*, *lacY*, and *lacA* genes after binding to the repressor. IPTG is a mimetic chemical of allolactose and exhibits good cellular permeability and low cytotoxicity. It is used as an inducer in most studies with the Lac operon. However, allolactose has about two times higher affinity for the repressor than IPTG [23]. Therefore, we thought that lactose, an isoform of allolactose, could bind to the repressor even though it has a weak binding affinity. To investigate whether GFP expression could be regulated with IPTG and lactose in mammalian cells, we transfected pCalo5-GFP as a control vector or pCalo5-GFP-LacI vectors into HEK293 cells. As a result, we observed that the cells transfected with pCalo5-GFP showed GFP expression. After transfection with pCalo5-GFP, HEK293 cells expressed GFP regardless of the induction time and IPTG and lactose concentrations (Fig. 2A). However, the cells transfected with pCalo5-GFP-LacI had almost no GFP expression without IPTG or lactose. After treatment with IPTG, HEK293 cells transfected with pCalo5-GFP-LacI showed weak GFP expression. However, no significant difference in the level of GFP expression was observed between HEK293 cells treated with 1 and 3 mM IPTG. To effectively induce GFP expression, IPTG should be used at a concentration of at least 1 mM for over 24 h (Fig. 2B). Although lactose could induce GFP expression at a very low level after 24 h in HEK293 cells, we observed that it could also induce GFP expression in mammalian cells (Fig. 2B). Thus, lactose has the potential to serve as an inducer in mammalian cells expressing the Lac operator.

### The Additive Effect of IPTG and Lactose To Elevate the Expression Level of GFP

**Fig. 3:**
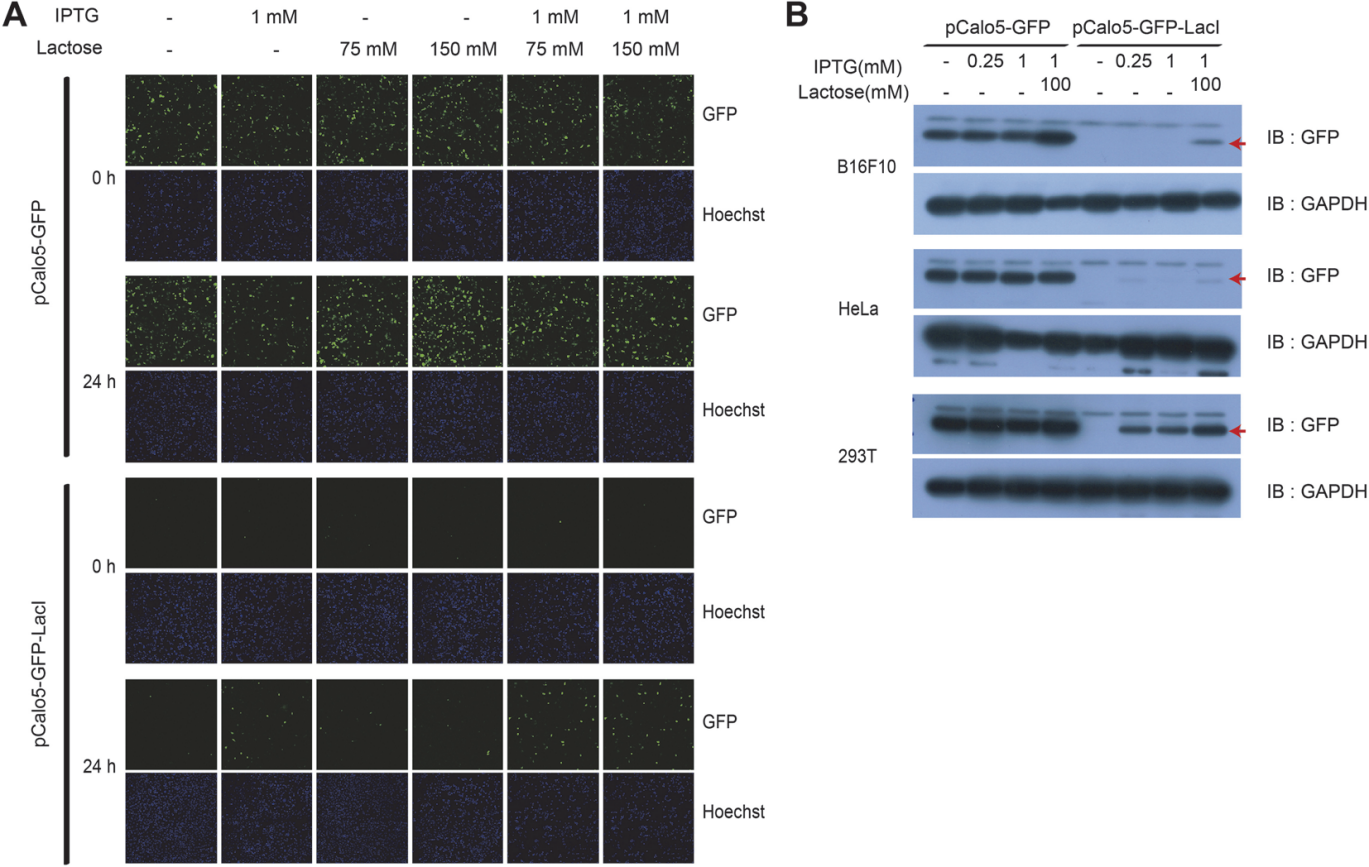
The increase of expression of GFP by co-treatment of IPTG and lactose. (**A**) HEK293 cells transfected with indicated vector were seeded into 24 well plates (1 × 105 cells/well) before induction of gene expression. After 8 h, various concentrations of IPTG and/or lactose were treated into transfected HEK293 cells for 0 h and 24 h. After indicated time, 10 μg/ ml of Hoechst were treated into each well for 10 min and 4% Paraformaldehyde were treated into each well for fixation of cells for 10 min. The green color represents expressed GFP in HEK293 cells and the blue color represents nucleus stained by Hoechst. (**B**) GFP expression can be regulated in other cell lines. Transfected B16F10, HeLa, and HEK293T cells were seeded into 24 well plates (1 × 105 cells/well). After 8 h, IPTG and/or lactose were treated into transfected cells for 24 h. After induction of GFP expression by IPTG and/or lactose, GFP expression level was analyzed by Western blot. GAPDH was used as control protein. Reactive bands were indicated by arrow.

IPTG irreversibly binds to the Lac repressor as an agonist of allolactose and induces the expression of target genes. The results in Fig. 2 show that IPTG alone was insufficient to induce the expression of GFP. IPTG and allolactose bind to the same position on the Lac repressor and allolactose has two times higher affinity for the promoter than IPTG. Lactose is an isoform of allolactose and could induce the expression of GFP, although the level of GFP expression was very low (Fig. 2B). The effect of lactose on the LacI repressor is unclear. We thought that lactose may exert an additive effect on the induction of GFP expression. We tested whether the co-treatment with IPTG and lactose could increase the efficiency of GFP expression. As a result, we found that the expression level of GFP was higher after co-treatment with IPTG (1 mM) and lactose (100 mM) than with IPTG alone after 24 h in HEK293 cells transfected with pCalo5-GFP-LacI (Fig. 3A). To quantitatively confirm these results, we carried out western blot analysis using cell lysates after 24 h of treatment with inducers, IPTG and/or lactose (Fig. 3B). We observed that GFP expression was stronger in B16F10, HeLa, and HEK293 cells transfected with pCalo5-GFP- LacI after co-treatment with IPTG and lactose than that observed after IPTG or lactose treatment alone (Fig. 3B). Thus, lactose could also improve gene expression in other mammalian cells expressing the Lac operon upon co-treatment with IPTG. It may also affect the inhibitory function of the Lac repressor in mammalian cells.

### The Optimum Concentration for the Efficient Induction of GFP Expression

**Fig. 4:**
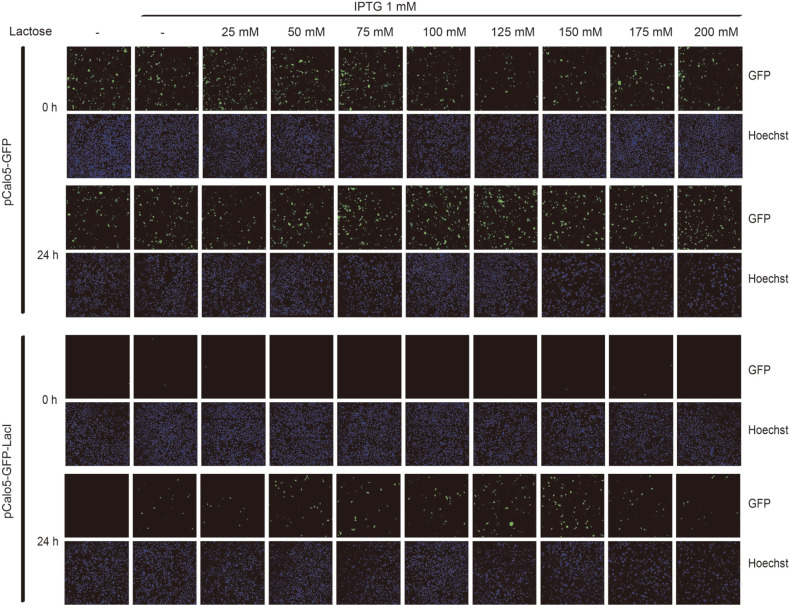
Optimal concentration for expression of GFP by IPTG and lactose in HEK293 cell line. HEK293 cells transfected with indicated vector were seeded into 24 well plates (1 × 105 cells/well) before induction of gene expression. After 8 h, indicated lactose with 1 mM of IPTG was treated into transfected HEK293 cells for 0 h and 24 h. After indicated time, 10 μg/ml of Hoechst was treated into each well for 10 min and 4% paraformaldehyde was treated into each well for fixation of cells for 10 min.

We observed that the co-treatment with IPTG and lactose could induce higher GFP expression than the treatment with IPTG or lactose alone. We confirmed the optimum concentration of inducers for the efficient induction of GFP expression with our inducible vector system. We simultaneously treated HEK293 cells with various concentrations of lactose and 1 mM of IPTG, which was the saturated concentration for the induction of GFP expression (Fig. 2B). We could observe that the expression level of GFP increased based on the concentration of lactose (Fig. 4). However, high concentration of lactose may cause osmotic shock and cell death. In our experiments, HEK293 cells began to die at lactose concentrations above 100 mM. We decided to use 75 mM as the optimum lactose concentration for the efficient expression of GFP because this concentration induced maximum GFP expression and caused minimum cell death (Fig. 4). We improved the efficiency of our inducible vector system by co-treatment with IPTG and lactose. However, this system still has low efficiency of gene expression, which needs to be addressed.

### The Deletion of Lac Operators for the Efficient Regulation of Gene Expression

**Fig. 5:**
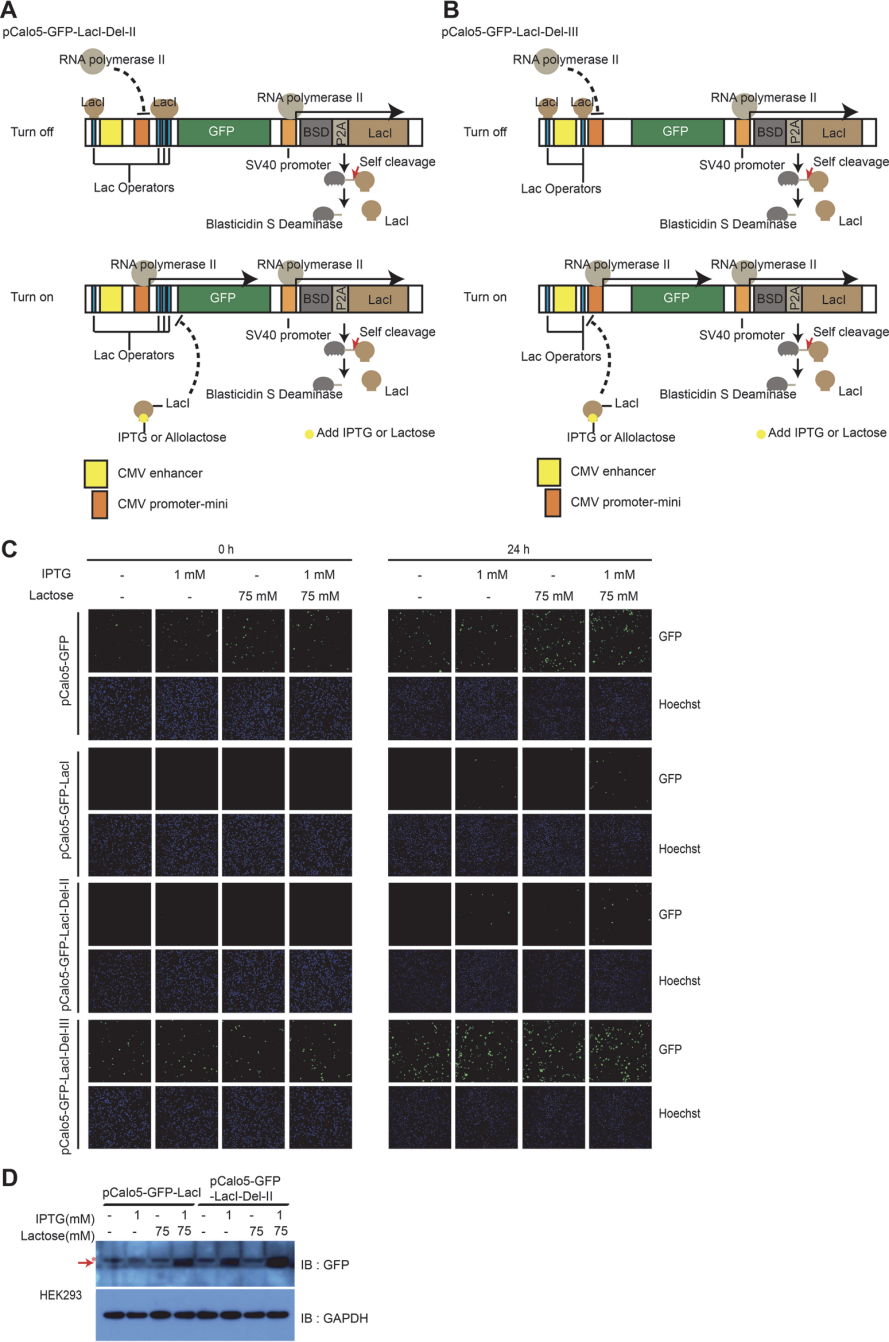
The efficiency of inducible vectors by co-treatment of IPTG and lactose in HEK293 cell line. The inducible vector, pCalo5-GFP-LacI-Del-II was modified from pCalo5-GFP-LacI by eliminate operator located between CMV promoter and enhancer. (**B**) The inducible vector, pCalo5-GFP-LacI-Del-III was modified from pCalo5-GFP-LacI by eliminate operators located between CMV promoter and GFP ORF. (**C**) HEK293 cells transfected with indicated vectors were seeded into 24 well plates (1 × 105 cells/well) before induction of gene expression by IPTG and/or lactose. After 8 h, various concentrations of IPTG and/or lactose were treated into transfected HEK293 cells for 0 h and 24 h. After indicated time, 10 μg/ml of Hoechst was treated into each well for 10 min and 4% Paraformaldehyde were treated into each well for fixation of cells for 10 min. (**D**) After induction of GFP expression by IPTG and/or lactose at the indicated concentrations for 24 h, GFP expression level was analyzed by western blot. GAPDH was used as control protein. Reactive bands were indicated by arrow, and asterisk indicated nonspecific bands.

The vector pCalo5-GFP-LacI has five operators and strongly silences the target gene expression. However, this vector system could induce only low level of GFP expression. Hence, we thought that the five operators massively block the binding of RNA polymerase II to CMV promoter-mini region through the LacI repressors, and significantly reduce the expression of GFP after inducer treatment. Thus, we constructed two modified inducible vectors by deleting the Lac operators from pCalo5-GFP-LacI vector to improve the expression level of our inducible vector system (Figs. 5A and B). We termed the resulting two vectors as pCalo5-GFP-LacI-Del-II and pCalo5-GFP-LacI-Del-III. The pCalo5-GFP-LacI-Del-II vector was constructed by the deletion of the Lac operator between CMV enhancer and CMV promoter-mini, while pCalo5-GFP-LacI-Del-III was derived after the deletion of three Lac operators between CMV promoter-mini and GFP gene.

We tested the efficiency of pCalo5-GFP-LacI-Del-II and pCalo5-GFP-LacI-Del-III vectors for regulating GFP expression in HEK293 cells. HEK293 cells transfected with pCalo5-GFP-LacI-Del-II vector showed complete abrogation of GFP expression after 24 h until being treated with inducers, and these cells expressed higher level of GFP than those transfected with pCalo5-GFP-LacI vector after 24 h of co-treatment with IPTG and lactose (Fig. 5C). The amount of GFP was measured by western blotting and the expression level was compared between HEK293 cell transfected with GFP-LacI-Del-II and GFP-LacI vectors. We confirmed that HEK293 cells transfected with GFP-LacI-Del-II vector hardly expressed any GFP before co-treatment with IPTG and lactose but expressed higher level of GFP than HEK293 cells transfected with pCalo5-GFP-LacI (Fig. 5D). However, the expression of GFP could not be regulated by inducers in HEK293 cells transfected with pCalo5-GFP-LacI-Del-III vector; continuous GFP expression was observed after transfection with pCalo5-GFP-LacI-Del-III vector (Fig. 5C). This observation could be attributed to the deletion of the three operators in the direction of RNA polymerase II to transcribe GFP. We showed the importance of locations and number of Lac operators; our results were consistent with those of previous studies [24-26]. Taken together, we demonstrated that the pCalo5-GFP-LacI- Del-II inducible vector system can be regulated with IPTG and lactose in mammalian cells and confirmed that lactose could serve as an inducer with IPTG and improve the efficiency of gene expression regulation in mammalian cells expressing the Lac operon system.

## Discussion

The development of tools for the regulation of important genes would reveal the functions and roles of several genes and could be useful for the treatment of genetic diseases. Many tools have been designed for the regulation of gene expression. Among these, the Lac operon system is used for the regulation of gene expression. IPTG is used as the inducer in the Lac operon system and offers great advantages. IPTG has high affinity for the LacI repressor (K_a_ = 10^6^ M^−1^) [22] and rapidly infiltrates into mammalian cells [23]. In addition, IPTG is stable at high temperatures and low pH [29]. These advantages have extended the application of the Lac operon system with mammalian cells. However, several tools based on the Lac operator-repressor system is a quite complicate expression system showing leaky expression of genes under non-inducing conditions [16] and may not dramatically turn on/off of the expression of genes. Here, we designed a simple inducible vector to address these issues. We show that the pCalo5-GFP-LacI vector tightly suppressed GFP expression under non-inducing conditions while inducing its expression in the presence of IPTG and/or lactose.

Jebe *et al.* (1973) showed the ability of lactose to bind to the LacI repressor and act as an anti-inducer in *E. coli* [30]. Lactose could bind to the LacI repressor at a concentration similar to that of IPTG under saturation conditions with a Ka value of 2 × 10^−4^ [30]. However, in the present study, we observed that lactose could also induce GFP expression and improve the induction efficiency of GFP expression in the presence of IPTG in mammalian cells. The tools using the Lac operon and repressor often show conflicting results with lactose between *E. coli* and mammalian cells. These differences may be associated with the variations in the infiltration ability of lactose into these cell types. Therefore, it is important to study the functions of lactose using the Lac operator and repressor in mammalian cells.

Although lactose failed to induce high level GFP expression in our inducible expression system, it could promote the expression of genes that may not be induced with IPTG. The regulation of important genes with lactose has great advantages. Lactose could be applied to other tools based on the Lac operator and repressor system to improve the induction efficiency of gene expression. Lactose is also easily available and the use of these tools to regulate the expression of genes with Lac operator and repressor is cost-effective.
